# Seroprevalence of antibodies to SARS‐CoV‐2 in healthcare workers in a nonepidemic region, Japan: A hospital‐based study on May, 2020

**DOI:** 10.1002/jmv.26962

**Published:** 2021-04-03

**Authors:** Akihiro Nakamura, Sanae Ando, Hideaki Endo, Ryoichi Sato

**Affiliations:** ^1^ Department of Cardiology Iwate Prefectural Central Hospital Morioka Iwate Japan; ^2^ Department of Clinical Laboratory Iwate Prefectural Central Hospital Morioka Iwate Japan

**Keywords:** antibody test, COVID‐19, Iwate, SARS‐CoV‐2, serosurveillance

## Abstract

The polymerase chain reaction (PCR) testing rate is low in our local area and the true rate of coronavirus disease 2019 (COVID‐19) infection may include many asymptomatic individuals. We conducted a serosurveillance using antibody testing in an area where official report of COVID‐19 infection is not done yet. Blood samples were obtained from 1404 healthcare workers (41 ± 11 years) in our hospital on May 29–31, 2020. First, the potential infection frequency was confirmed using two quantitative antibody tests. In addition, the usefulness of rapid antibody kit testing for COVID‐19 serosurveillance was examined. A COVID‐19‐indected case was defined as showing positive results in both quantitative tests. None of 1404 samples had positive results from the two quantitative tests. The false positive rates were 0.36% and 0.07%, whereas those in rapid antibody kits were 3.3% and 3.0%. In conclusion, as of May, 2020, potential spread mainly by asymptomatic individuals infected with COVID‐19 was not found in our local area where there was no official report of COVID‐19, even if the PCR testing rate was low. Rapid antibody kits might not be useful due to the high false positive rate in an area with a low incidence of COVID‐19 infected individuals.

## 1 INTRODUCTION

Coronavirus disease 2019 (COVID‐19) caused by the severe acute respiratory syndrome coronavirus 2 (SARS‐CoV‐2) has spread rapidly worldwide and affected human health and social life.^1^, ^2^, ^3^ In Japan, the first COVID‐19 case was confirmed in January 16, 2020. After the first peak of the epidemic on April 11, the number of COVID‐19 cases gradually decreased and returned to January's early onset levels. As of October 1, 2020, the total number of cases had reached 84,335.^4^ Among the 47 prefectures of Japan, only Iwate, Japan's northeastern prefecture with a population of 1,227,647 individuals (as of April 2020), had no reported COVID‐19‐infected cases until July 28, 2020, and the latest official number of cumulative cases is 23 as of October 1, 2020.^5^ As of May 29, 2020, over 6000 inquiries for the polymerase chain reaction (PCR) testing had been made to a local hotline from concerned individuals, however, Iwate conducted only 730 tests, which were the lowest level in Japan.^5^ From the limited PCR testing, the true COVID‐19‐infected rate may include many asymptomatic individuals.

Iwate Prefectural Central Hospital is in Morioka, the capital city of Iwate and plays a central role in disease treatment and prevention with providing high‐level medical research and services for the region's population. An orthogonal testing with two or more quantitative antibody tests with a very high specificity (99.5% or greater) has been useful in populations with a very low prevalence of COVID‐19.^6^ This study was designed to find out the potential spread mainly by asymptomatic individuals infected with COVID‐19 in our local area. For this primary purpose, we conducted an orthogonal quantitative antibody testing with blood samples from healthcare workers in our hospital. In addition, the usefulness of rapid antibody kits for point‐of‐care (POC) was examined.

## 2 MATERIALS AND METHODS

### 2.1 Study design

**Figure 1:**
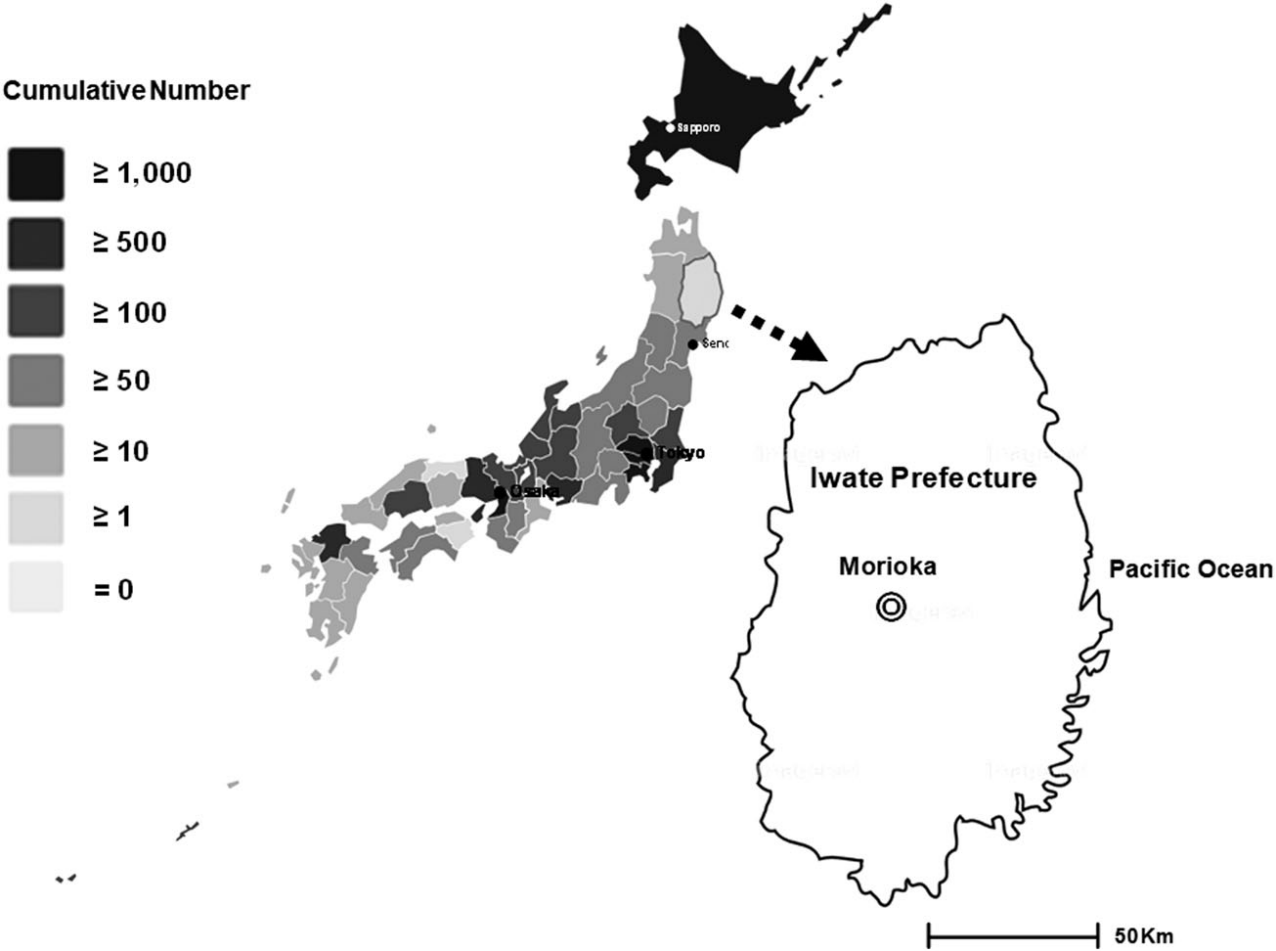
The location of Iwate Prefecture and Iwate Prefectural Central Hospital. Number of COVID‐19 cases by prefecture on May 31, 2020, based on the statistics from the Japanese Ministry of Health, Labor and Welfare^4^

Iwate Prefecture, with 1.2 million residents, is on the Pacific coast of northeastern Japan. To determine the seroprevalence of COVID‐19 in our region, we retrospectively evaluated COVID‐19 antibodies in serum from healthcare workers at Iwate Prefectural Central Hospital in the city of Morioka (Figure 1). The hospital, which has 685 beds, with an average daily number of 1100 outpatients and 534 inpatients in 2019, is one of the core medical institutions in Iwate Prefecture. The study protocol was approved by the ethics committee of Iwate Prefectural Central Hospital, Iwate, Japan (approval number, 343), and carried out according to the principles of the Declaration of Helsinki.

### 2.2 Study population and antibody tests

Blood samples were taken during the annual health checkups of 1706 healthcare workers (physicians, nurses, pharmacists, radiographers, laboratory technicians, and medical office workers) on May 18–29, 2020, and stored at −20°C. Serum samples (*n* = 1404) from employees with informed consent were analyzed for the detection of antibodies to COVID‐19 using the laboratory‐based quantitative and POC qualitative tests on May 29–31, 2020.

Two laboratory‐based quantitative tests that were approved by the US Food and Drug Administration were used: Abbott Architect® SARS‐CoV‐2 IgG Assay (chemiluminescent microparticle immunoassay; sensitivity, 100%; specificity, 99.6%) (Abbott Laboratories, Abbott Park)^7^ and Roche Elecsys® Anti‐SARS‐CoV‐2 RUO Assay (electrochemiluminescent immunoassay; sensitivity, 100%; specificity, 99.8%) (Roche Diagnostics).^6^ Two rapid antibody kits for POC were performed with Instant‐view® plus COVID‐19 Test: Alfa test (lateral flow chromatographic immunoassay; sensitivity, 97.8%; specificity, 94.6%) (Alfa Scientific Designs), and Cellex qSARS‐CoV‐2 IgG/IgM Rapid Test: Cellex test (lateral flow chromatographic immunoassay; sensitivity, 93.8%; specificity, 95.6%) (Cellex, Research Triangle Park). All tests were conducted at room temperature and according to each manufacturer's instructions. The results were read visually after 10 min.

A case was considered to be infected with COVID‐19 only if it tested positive in both laboratory‐based quantitative tests; a sample receiving only one positive result was considered noninfected. The prevalence of COVID‐19 was determined by dividing the number of infected cases by that of samples. Continuous variables were expressed as mean ± *SD*, and categorical variables were expressed as numbers and percentages. The 95% confidence intervals (95% CIs) for the positive results in the tests were presented by the Wald method using Microsoft Excel.

## 3 RESULTS

**Figure 2:**
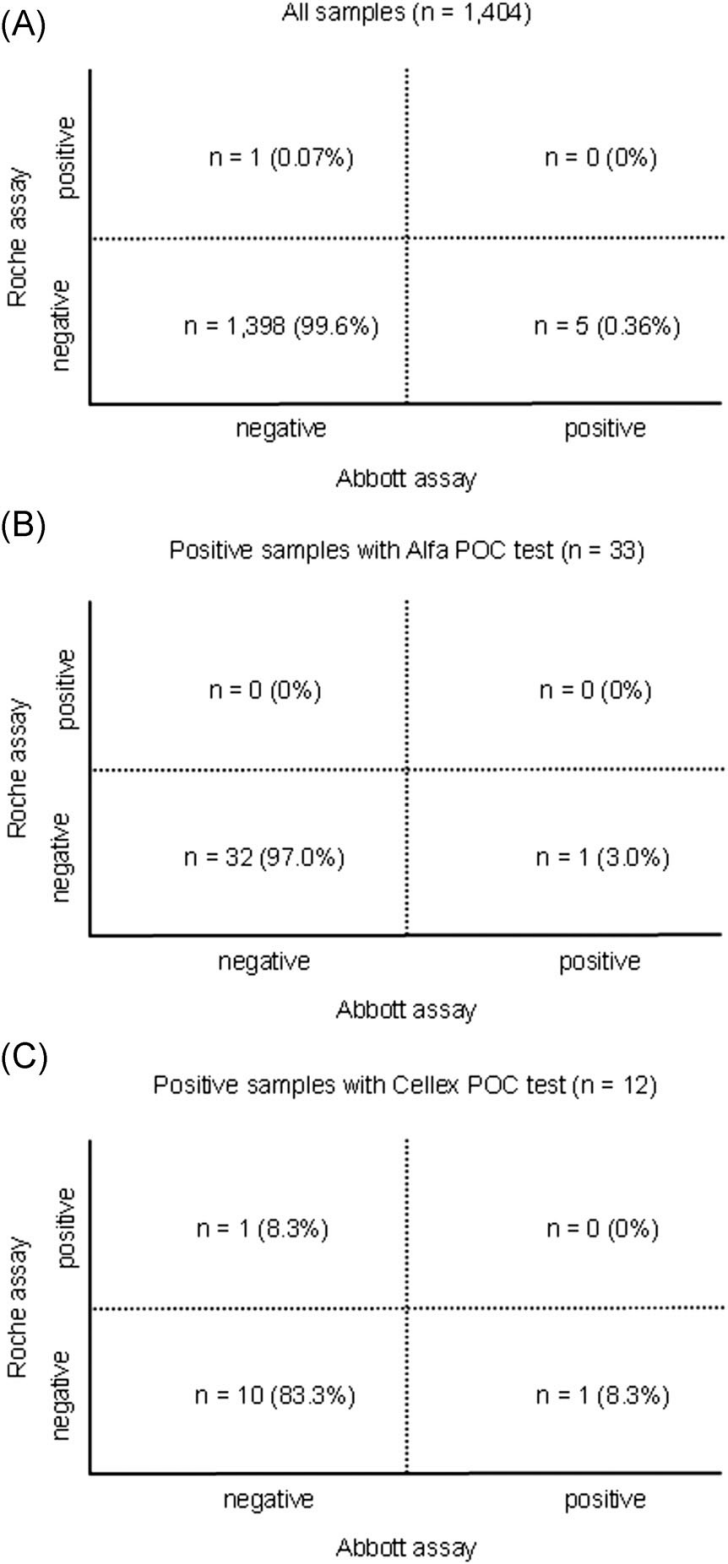
Orthogonal comparison of Abbott's and Roche's assays in all samples (*n* = 1404) (A) and samples with positive for rapid antibody kits: Alfa POC test (*n* = 33) (B); Cellex POC test (*n* = 12) (C)

A total of 1404 serum samples (386 men and 1018 women; mean age, 41 ± 11 years) were analyzed in this study. The laboratory‐based quantitative tests detected positive results in 6 of 1404 samples (0.43%) (95% CI, 0.09–0.77): Abbott's test in five samples (0.36%) (95% CI, 0.05–0.67) and Roche's test in 1 (0.07%) (95% CI, −0.07 to 0.21). According to the study design that required positive results in both tests, no cases were confirmed positive (Figure 2A).

All samples were also analyzed with the rapid antibody kits for POC: 1000 samples with Alfa's test; 404 samples with Cellex's test. When compared with the laboratory‐based quantitative tests, both rapid antibody kits for POC had higher rates of positive results in 33 of 1000 samples (3.3%) (95% CI, 2.19–4.41) with Alfa test; 12 of 404 samples (3.0%) (95% CI, 1.34–4.66) with Cellex test. No sample had a positive result in both laboratory‐based quantitative tests and rapid antibody kits for POC (Figures 2B,C).

## 4 DISCUSSION

To the best of our knowledge, this is the first study to evaluate the potential rate of antibodies to COVID‐19 in a region having no officially confirmed cases of COVID‐19 infection. We obtained blood samples from healthcare workers in our hospital, and quantitatively measured COVID‐19 antibodies in serum using two laboratory‐based immunoassays tests (Abbott's and Roche's tests). The results showed that there were no positive immune responses detected in both laboratory‐based quantitative tests. Additional findings were that the rapid antibody kit testing might not be helpful for COVID‐19 serosurveillance due to the high false positive response rates in an area with a very low incidence of COVID‐19‐infected individuals.

The serological tests to detect COVID‐19 antibodies are crucial for knowing infection status individually and monitoring the spread of the virus. In Japan, the positive rate of COVID‐19 antibodies was 3.3% in 1000 outpatients who visited Kobe City Medical Center General Hospital.^7^ Blood samples obtained from 202 patients at community clinics in Tokyo showed a 5.9% rate of infection.^8^ The COVID‐19 antibodies survey by the Japanese Ministry of Health, Labor and Welfare showed only a 0.03% (1 of 3009 samples) positive rate in Iwate's neighboring prefecture of Miyagi.^4^ These data and our results suggest a low prevalence of COVID‐19 in the Tohoku district compared with the rates in Tokyo and Kobe City, even if the PCR testing rate was low.

Several commercial COVID‐19 antibody immunoassays are now available. In a region with very low prevalence, an antibody test with a high specificity (perhaps ≥ 99.5%) should be used, which would yield a higher positive predictive value.^9^ This study's laboratory‐based quantitative tests had a high specificity (99.6% and 99.8% in Abbott's and Roche's assays, respectively), and positive results were only detected in four (0.36%) samples in the former and one (0.07%) sample in the later. In the rapid antibody kits for POC, there were more false‐positive COVID‐19 antibody results, whose specificity were relatively low (94.6% and 95.6%). Although the rapid qualitative kits are reliable in areas with a high‐prevalence of COVID‐19,^10^ they have the potential for poor accuracy in low ‐prevalence area. The antibody tests with high accuracy and consistent performance are needed not only in Iwate Prefecture but also throughout Japan to determine the prevalence of COVID‐19.

This study has several limitations. First, it was a survey from a single medical institution with a relatively small number of blood samples. The participants in this study were healthcare workers; thus, younger or older individuals were not enrolled. Second, the positive and negative control tests are significant for the full validation of COVID‐19 antibodies and assessment of nonspecific binding. In this study, attempts to use the positive or negative control test presented difficulties because of no identified infected cases and the stay‐at‐home order that affected the population. Third, the timing of COVID‐19 antibody tests may be significant for accurate COVID‐19 antibody detection. Because recent reports showed the disappearance of antibody or a decrease in the titer after COVID‐19 infection,^11^, ^12^ a repeated measurement of antibody would be warranted after a potent epidemic.

## 5 CONCLUSION

The seroprevalence of the positive COVID‐19 antibodies was 0% in a retrospective study with the blood samples from our hospital's healthcare workers in Iwate, where there was no official report of COVID‐19. This results shows no potential spread primarily by subclinical infected cases in our area. Laboratory‐based quantitative tests with a high specificity, but not rapid antibody kits for POC with a poor specificity, are required for antibody serosurveillance in an area with a low prevalence of COVID‐19 cases.

## AUTHOR CONTRIBUTIONS

*Study design*: Akihiro Nakamura, Sanae Ando, Hideaki Endo, and Ryoichi Sato. *Performance of study*: Akihiro Nakamura, Sanae Ando, and Ryoichi Sato. *Data analysis*: Akihiro Nakamura and Hideaki Endo. *Wrote, reviewed, and edited manuscript*: Akihiro Nakamura, Sanae Ando, Hideaki Endo, and Ryoichi Sato.

## CONFLICT OF INTERESTS

The authors declare that there are no conflicts of interests.
